# Prospective comorbidity-matched study of Parkinson's disease and risk of mortality among women

**DOI:** 10.1136/bmjopen-2016-011888

**Published:** 2016-09-23

**Authors:** Anke C Winter, Pamela M Rist, Julie E Buring, Tobias Kurth

**Affiliations:** 1 Division of Public Health Sciences, Department of Surgery, Washington University School of Medicine, St. Louis, Missouri, USA; 2 Division of Preventive Medicine, Department of Medicine, Brigham and Women's Hospital, Harvard Medical School, Boston, Massachusetts, USA; 3 Institute of Public Health, Charité-Universitätsmedizin, Berlin, Germany

**Keywords:** EPIDEMIOLOGY, mortality

## Abstract

**Background:**

Individuals with Parkinson's disease (PD) may have an increased risk of overall mortality compared to the general population. Women may have lower mortality rates from PD than men; however, studies among women on the effect of PD on mortality have been limited and may not have adequately controlled for confounding by comorbidities.

**Methods:**

We conducted a matched cohort study among participants in the Women's Health Study. 396 incident PD cases were identified through self-report. Each PD case was matched by age to a comparator who was alive and had the same modified Charlson comorbidity score as the PD case. The PD cases and matched comparators were followed for all-cause mortality. Cox proportional hazards models adjusted for age at the index date, smoking, alcohol consumption, exercise and body mass index were used to determine the association between PD and mortality.

**Results:**

During a median of 6.2 years of follow-up, 72 women died (47 PD cases and 25 comparators). The multivariable-adjusted HR for mortality was 2.60 (95% CI 1.56 to 4.32).

**Conclusions:**

PD was associated with more than a twofold increased risk of all-cause mortality among women. Results are similar to those observed among men.

**Strengths and limitations of this study**

- We performed an age-matched and comorbidity-matched cohort study to determine the association between Parkinson's disease (PD) and mortality among women.
- Only incident cases of PD were used and deaths were confirmed by medical record review.
- Our PD cases were based on self-reports of physician diagnosis and we did not have information on the Hoehn and Yahr stage or other clinical information on severity of PD.
- Results on the association between PD and specific causes of death are limited due to the low number of outcome events, and should be interpreted with caution.

## Introduction

Parkinson's disease (PD) is a progressive neurodegenerative movement disorder characterised by its cardinal features of tremor, bradykinesia, rigidity and postural instability. The incidence of PD increases with age with an estimated overall incidence rate of 17 per 100 000 person-years.^1^ Men have higher incidence rates compared to women, particularly between the ages of 60 and 80 years.^2^ There is increasing evidence that gender disparities are not limited to PD incidence but are also relevant in the context of PD symptoms, treatment and disease prognosis.^3^

PD has been linked to an increased risk of overall mortality compared to the general population.^4^ ^5^ Among men, PD is associated with an approximately twofold increase in the risk of all-cause mortality.^5^ It has been hypothesised that women may have lower mortality rates from PD than men.^3^ However, the evidence comparing mortality rates according to gender is inconclusive. The majority of studies indicate that women may have lower mortality rates compared to men,^6–13^ but some studies suggest higher mortality rates among women.^14–17^ Many prior studies, which estimated mortality risks among females with PD, have been small and between-study heterogeneity has impeded pooling effect estimates.^4^ Additionally, these studies may not have adequately controlled for confounding by comorbidities, and often included prevalent cases of PD. Given the limited evidence and limitations of previous studies, further studies are warranted to evaluate the association between PD and mortality risk among women and help elucidate possible gender disparities in relation to PD mortality.

We therefore performed an age-matched and comorbidity-matched cohort study among participants in the Women's Health Study (WHS) to determine whether PD may increase the risk of mortality among women. By using only incident cases of PD, performing comorbidity-matching and having a large underlying cohort, our study has several strengths compared to previous research.

## Methods

The WHS was a randomised, placebo controlled trial of the effects of low-dose aspirin and vitamin E in the primary prevention of cardiovascular disease and cancer among 39 876 women.^18^ ^19^ After the end of the trial in 2004, women were asked if they would be willing to continue to be followed on an observational basis. Observational follow-up is currently ongoing.

Women have received yearly questionnaires asking about demographic, lifestyle and health information, including physician-diagnosed PD. We identified 398 women who self-reported ‘yes’ to the question on physician-diagnosed PD. Next, to balance the age and comorbidity burden of PD cases and comparators, we attempted to match each PD case to a comparator following previously developed methods.^20^ Briefly, a participant was eligible to be a comparator if she was free from PD at the time the case was diagnosed (the index date), had the same age and same modified Charlson comorbidity score as the case on the index date, and did not report PD during the 5 years after the index date (to avoid preclinical cases being included in the comparator group). However, we allowed the selection of comparators who died within the first 5 years after the index date without reporting PD. The modified Charlson comorbidity index, which reflects accumulated comorbidity status (including myocardial infarction, congestive heart failure, peripheral vascular disease, dementia, chronic pulmonary disease, connective tissue disease, ulcer disease, liver disease, diabetes, renal disease, tumours, leukaemia, lymphoma, multiple myeloma and AIDS) between study entry and index date (ie, the date of reported physician-diagnosed PD), was calculated using self-reported health information according to previously developed methods.^20^ ^21^ Of the 398 self-reported PD cases, we were able to match 396 PD cases to comparators.

Our outcome of interest was all-cause mortality. Mortality follow-up is over 99% complete in the WHS. Deaths were identified by reports from family members or next of kin, postal authorities and searches of the National Death Index. Information on date and cause of death was confirmed through review of death certificates and medical records by an end points committee of physicians. Only confirmed causes of death were included in our analysis.

PD cases and matched comparators were followed from the date of diagnosis of the PD case to death, loss to follow-up or the end of the study (31 December 2013), whichever occurred first. Characteristics at baseline (1993–1995) of the PD cases and comparators were described using means for continuous variables and proportions for categorical variables. Mean duration of PD was calculated from PD onset to death, loss to follow-up or end of study. We used Cox proportional hazards models to calculate the HR between PD and death. Since age is a strong risk factor for death, we used age instead of time-on-study as our time scale for these analyses.^22^ Model one adjusted for age at the index date (in years), Charlson comorbidities score at the index date (continuous) and smoking status at baseline in WHS (never, past and current). Although we matched on age and Charlson comorbidity score at index date, when additional covariates are included in the model, the matching factors also need to be adjusted for to obtain unbiased effect estimates.^23^ Model two additionally adjusted for baseline alcohol consumption (rarely/never, 1–3 drinks/month, 1–6 drinks/week, ≥1 drink/day), exercise (rarely/never, <1 time/week, 1–3 times/week, ≥4 times/week) and body mass index (<25 kg/m^2^, 25 to <30 kg/m^2^, ≥30 kg/m^2^) as measured at enrolment into WHS. Three women were missing information on smoking status and were assigned to the reference category (never smoker) and four women were missing information on BMI and were assigned to the reference category (<25 kg/m^2^).

We explored effect modification by smoking status (never vs ever smoking), disease duration (<5 years vs ≥5 years) and age on PD onset (<70 vs ≥70 years of age) by stratification. To test for statistically significant effect modification, we included an interaction term in the model and used a χ^2^ test to determine if the interaction term was statistically significant. All models adjusted for age and models for disease duration and age at onset were additionally adjusted for smoking status. We used a competing risk Cox model adjusted for age and smoking status to analyse the association between PD and three competing causes of death: cardiovascular disease, cancer or other illnesses. We considered a two-tailed p<0.05 as statistically significant.

## Results

The baseline characteristics of PD cases and age-matched and comorbidity-matched controls are presented in table 1. As expected, baseline characteristics of PD cases and comparators were similar, although PD cases were less likely to report being a current or past smoker at enrolment in the WHS.

**Table 1:** Baseline characteristics of PD patients and age- and comorbidity- matched comparators

	PD patients n=396	Comparators n=396
Mean age at randomization, years (SD)	58.7 (8.0)	58.7 (8.0)
Mean age at PD diagnosis, years (SD)	70.6 (8.8)	n.a.
Mean duration of PD, years (SD)	6.7 (4.5)	n.a.
History of smoking, n (%)		
Never	241 (60.9)	191 (48.2)
Past	129 (32.6)	159 (40.2)
Current	26 (6.6)	46 (11.6)
Alcohol use, n (%)		
Rarely/never	201 (50.8)	172 (43.4)
1-3 drinks/month	54 (13.6)	48 (12.1)
1-6 drinks/week	99 (25.0)	139 (35.1)
≥1 drink/day	42 (10.6)	37 (9.3)
Physical activity, n (%)		
Rarely/never	180 (45.5)	149 (37.6)
<1/week	62 (15.7)	76 (19.2)
1-3 times/week	119 (28.5)	119 (30.1)
≥4 times/week	41 (10.4)	52 (13.1)
BMI (kg/m^2^), n (%)		
<25	199 (50.6)	203 (51.4)
25-<30	131 (33.3)	120 (30.4)
≥30kg/m^2^	63 (16.0)	72 (18.2)
Mean Charlson comorbidity score, at index date (SD)	1.18 (1.44)	1.18 (1.44)

BMI, body mass index; NA, not applicable; PD, Parkinson's disease.

**Figure 1:**
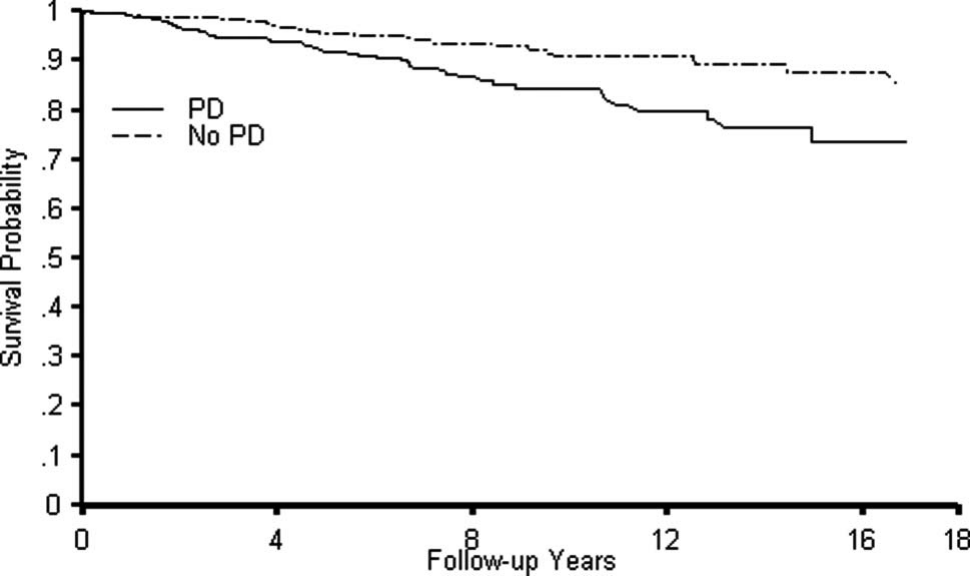
Kaplan Meier curves for the survival probability among individuals with Parkinson's disease (PD) and matched comparators.

During a median of 6.2 years of follow-up, 72 women died (47 PD cases and 25 comparators). The Kaplan-Meier curve for overall survival is displayed in figure 1 and shows that the PD cases have increased risk of death compared to the comparators (p value<0.01). The age-adjusted and smoking-adjusted HR for mortality was 2.65 (95% CI 1.60 to 4.37) and the multivariable-adjusted HR was 2.60 (95% CI 1.56 to 4.32). An exploratory analysis observed that the HR was higher among those who were never smokers (HR=4.01; 95% CI 1.81 to 8.86) than ever smokers (HR=1.75; 95% CI 0.88 to 3.45); however, the interaction between smoking and PD did not reach statistical significance (p value=0.10). We observed higher HRs among those with a longer disease duration (≥5 years) (HR=3.43; 95% CI 1.58 to 7.44) than shorter disease duration (<5 years) (HR=1.57; 95% CI 0.78 to 3.17); however, the interaction between disease duration and PD was not statistically significant (p value=0.12). The association between PD and mortality was slightly higher for those <70 years of age (HR=3.44; 95% CI 1.43 to 8.23) than those ≥70 years of age (HR=2.21; 95% CI 1.19 to 4.11); however, the interaction between age at onset and PD was not statistically significant (p value=0.32).

In exploratory analyses, PD was associated with an increased risk of death from cardiovascular disease (HR=2.65; 95% CI 0.98 to 7.19), cancer (HR=1.59; 95% CI 0.59 to 4.26), and other causes (HR=3.50; 95% CI 1.67 to 7.34).

## Discussion

In this age-matched and comorbidity-matched cohort study among women, PD was associated with a more than twofold increased risk of all-cause mortality. Results are similar to those observed in a study among men which also performed age matching and comorbidity matching^20^ (HR=2.32; 95% CI 1.85 to 2.92). This suggests that the effect of PD on risk of mortality is similar in men and women.

Heterogeneity across studies enrolling women has prevented the pooling of effect estimates from prior studies.^4^ Effect estimates ranged from 1.01 to 3.64, with 12 of the 17 studies finding less than a twofold increase in the risk of mortality among those with PD. The current study was larger than many prior studies and used comorbidity matching to control for confounding by the presence of other diseases. Confounding by comorbidities may result in an underestimate of the true effect of PD on mortality. For example, some studies have suggested that individuals with PD are less likely to have a prior diagnosis of most cancers than individuals without PD.^24–26^ Lack of adequate control for cancer comorbidities would result in an underestimate of the effect of PD on mortality. Similarly, smokers are less likely to develop PD,^27^ but smoking is linked with several other comorbidities. A higher prevalence of smoking-related comorbidities among the matched comparators without PD may also result in an underestimate of the true effect of PD on mortality. Thus, the higher effect estimate observed in this study may be partially due to the improved methods of controlling for confounding by comorbidities used in this study.

We observed some evidence that smoking status may modify the effect of PD on mortality risk with the strongest effect of PD on mortality being seen among never smokers, which has also been observed in some,^14^ ^28^ but not all,^20^ ^29^ prior studies.

Some limitations to our study should be noted. Identifying PD, particularly in early disease stages, is challenging in clinical and research settings and we cannot rule out potential misclassification.^30^ Our PD cases were based on self-reports of physician diagnosis and we did not have information on the Hoehn and Yahr stage or other clinical information on severity of PD. However, self-reported PD diagnoses have been shown to be highly valid among male health professionals.^20^ ^31^ Furthermore, participants who reported PD within 5 years of the index date were excluded to reduce the possibility of having participants with subclinical PD in our comparator group. Our cohort was composed of female health professionals who are primarily white, which may limit the generalisability of our findings to other racial or ethnic groups or to populations of different socioeconomic status. Additionally, our results may not be generalisable to populations with younger ages at PD onset since the average age at PD onset in our population was 70 years of age. Although we employed several methods to control for confounding, residual confounding may still be present. Our study design is a prospective comorbidity-matched study and some general limitations inherent to the matching procedure may also apply to our study. For example, by matching, we restricted our study population to those individuals who could be matched based on their age and comorbidity profile. This could potentially reduce the external validity of our study. However, only 2 of our 398 PD cases could not be matched in our study, so we do not expect a large impact on the generalisability of our results. Our analyses of potential effect modification by smoking or age at onset should be interpreted with caution given the low power to detect effects in strata and the possibility of false-positive findings when several subgroups are analysed.^32^ Finally, results on the association between PD and specific causes of death are limited due to the low number of outcome events, and should be interpreted with caution.

Our study has several strengths including the prospective design, large sample size, identification of incident PD cases and collection of a large amount of covariate information that allowed us to perform comorbidity matching as well as control for other lifestyle variables. We carefully adjusted for age, a strong predictor of both PD onset and mortality, by matching on age, using age as the primary time scale in our analyses and including it as a covariate in all analyses. Finally, all deaths were confirmed by review of medical records.

In conclusion, we observed that PD was associated with an approximately twofold increase in the risk of mortality among women in this population-based comorbidity-matched cohort study.
